# Supplementary figures and images for: GLAMbox: A Python toolbox for investigating the association between gaze allocation and decision behaviour
Source: PLoS One. 2019 Dec 16;14(12):e0226428. doi: 10.1371/journal.pone.0226428 (PMC6914332; doi:10.1371/journal.pone.0226428)

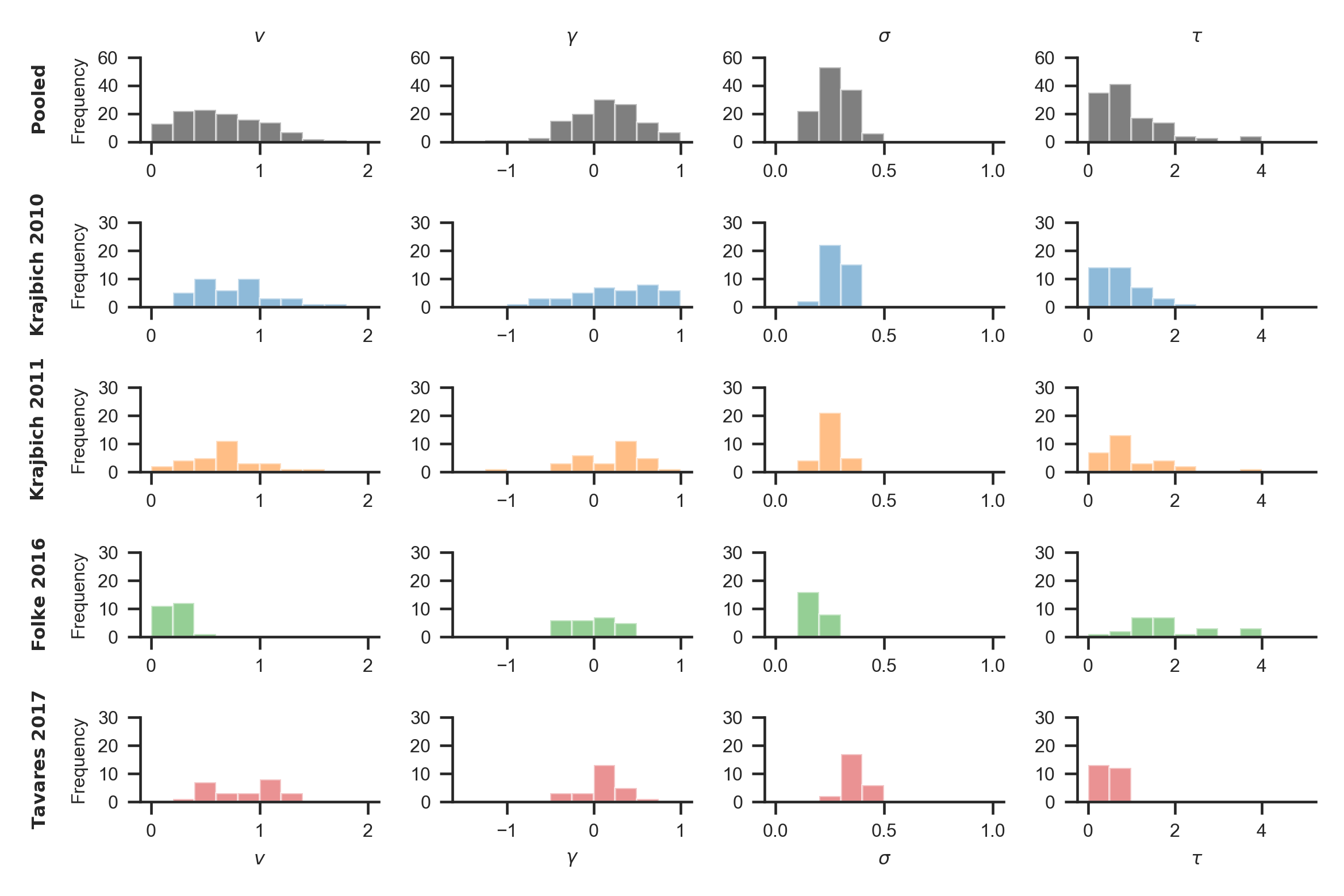

Supplement: S1 Fig — The top row contains distributions of parameter estimates across datasets. Subsequent rows show distributions per dataset: Krajbich et al. (2010; blue), Krajbich & Rangel (2011; orange), Experiment 2 from Folke et al. (2017; green) and Experiment 1 from Tavares et al. (2017; red). (TIFF) [file pone.0226428.s001.tiff]

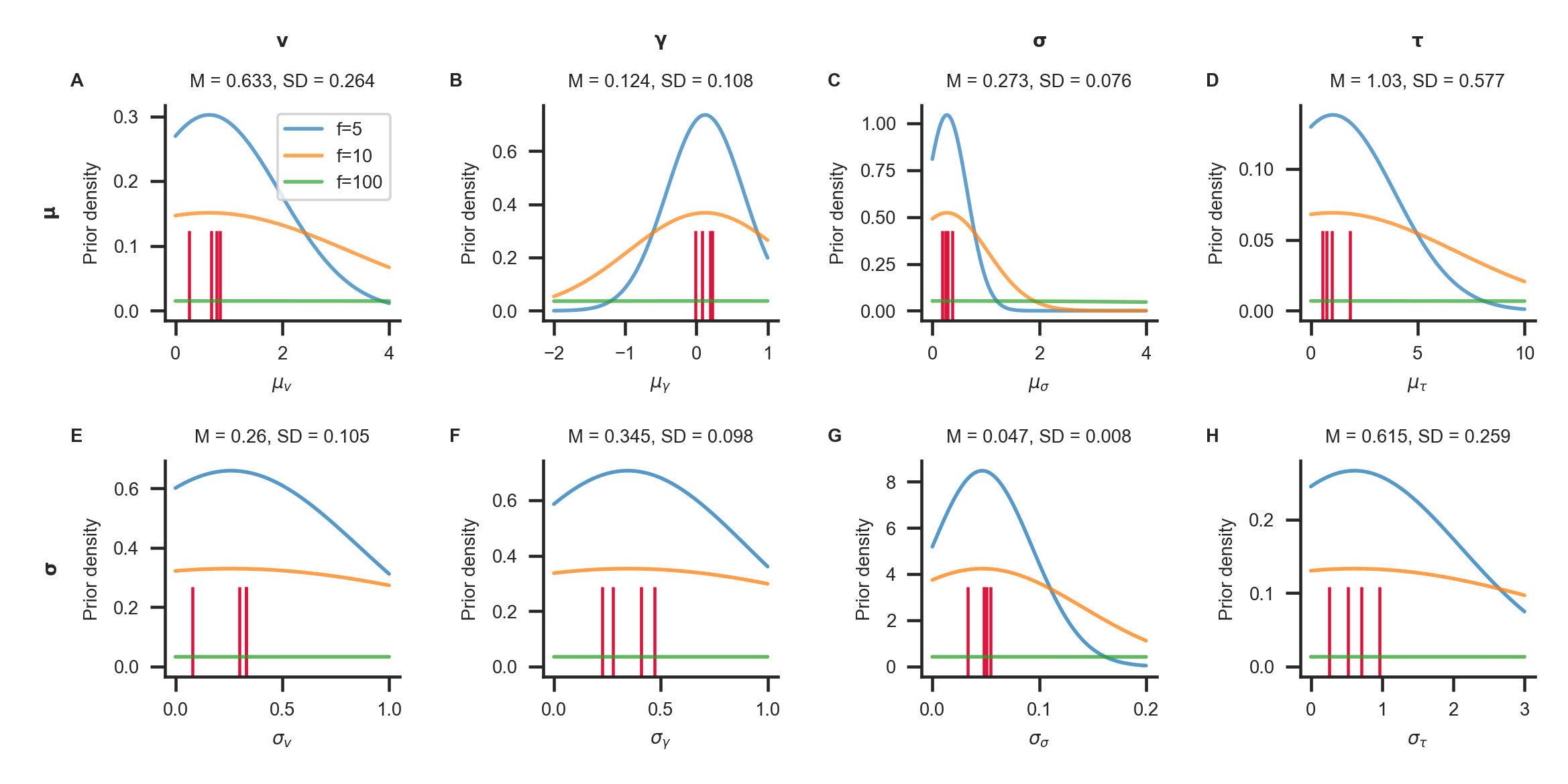

Supplement: S2 Fig — Different hyperpriors based on group-averaged parameter values were obtained from fitting the model to four different datasets (Folke et al., 2017; Krajbich et al., 2010; Krajbich & Rangel, 2011; Tavares et al., 2017; see S1 Table and S1 Fig). Panels show prior distributions on group level mean (upper row) and standard deviation (lower row) for each model parameter (columns; from left to right: v, γ, σ, τ). Observed group level estimates from the four datasets are indicated as red ticks in each panel. Blue, orange and green lines represent prior distributions with increasing levels of vagueness f. They are constructed as normal distributions with mean equal to the mean of the observed group level parameters across datasets (M), and standard deviation equal to f times the observed standard deviation across datasets (SD). Higher values of f correspond to wider, less informative priors. Prior distributions are further bounded between sensible limits. The user can specify the factor f during specification of hierarchical models. By default, hyperpriors with f = 10 (orange lines) are used. (TIFF) [file pone.0226428.s002.tiff]

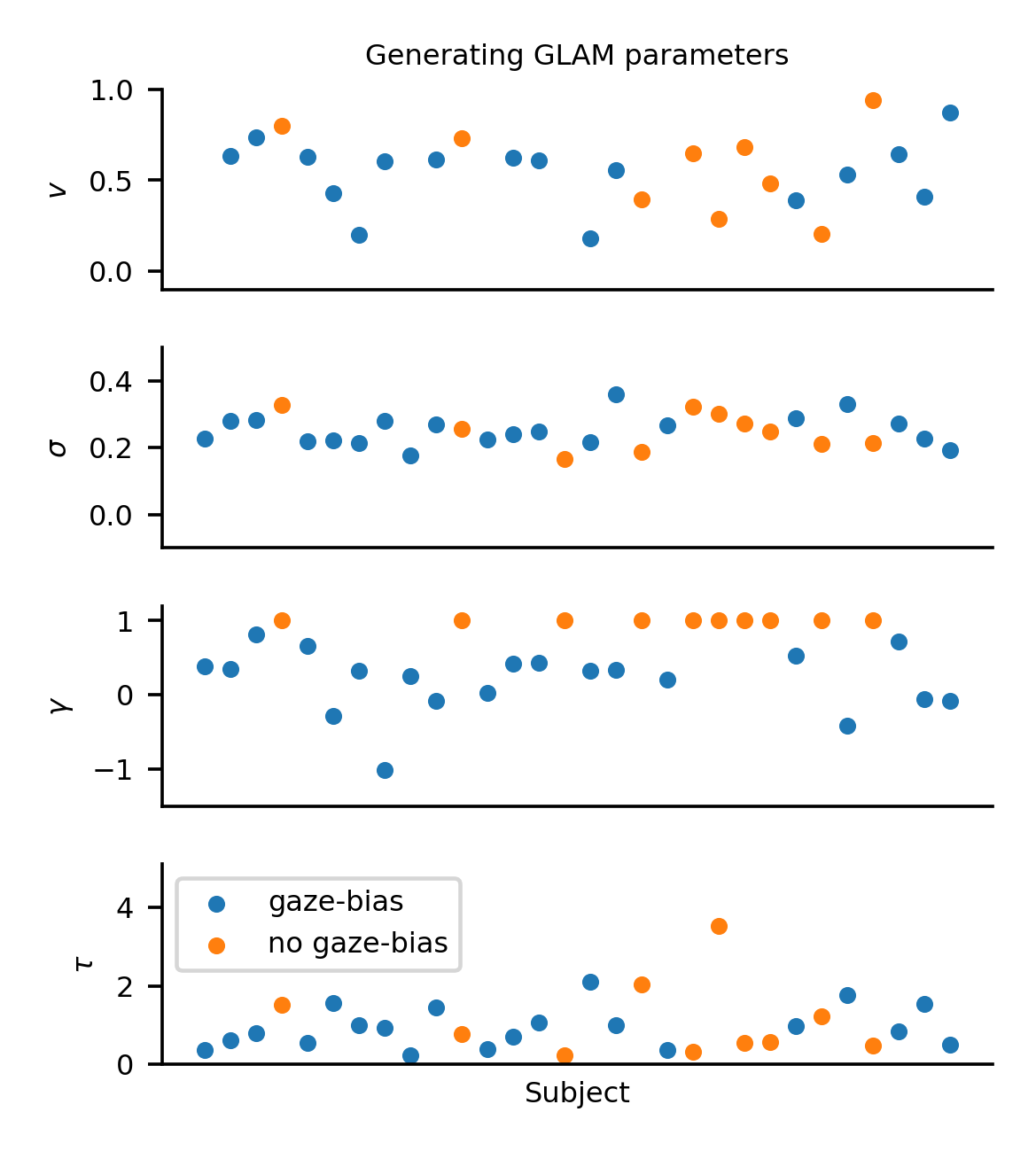

Supplement: S3 Fig — Colours indicate whether a subject was simulated with or without gaze bias. (TIFF) [file pone.0226428.s003.tiff]

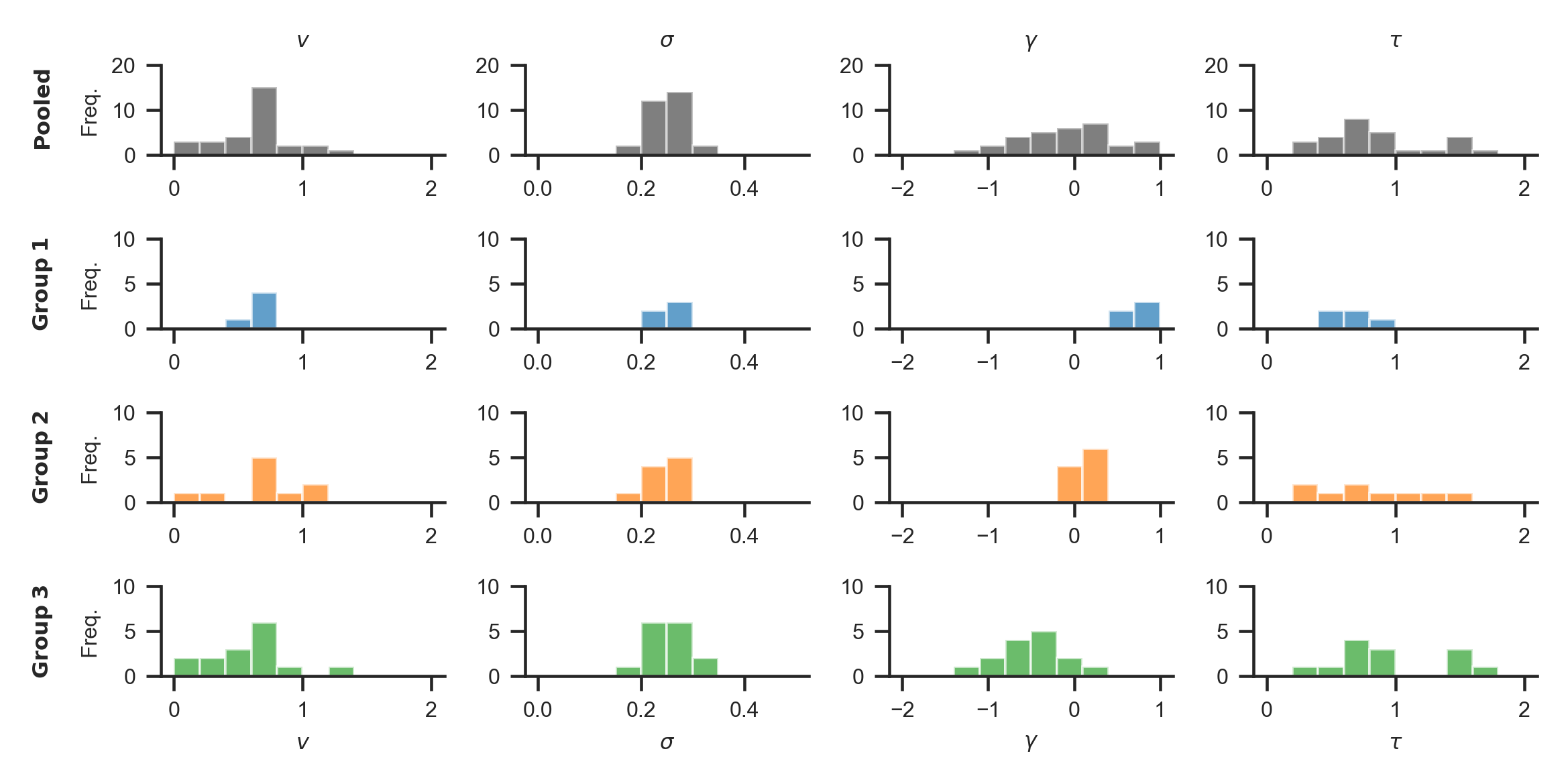

Supplement: S4 Fig — The top row shows distributions pooled across groups. The bottom three rows show distributions per group. Note that the groups do not differ systematically with respect to the velocity parameter v, the noise parameter σ, or the scaling parameter τ (first, second and last column; even though there is some variability between individuals). The groups differ, however, on the gaze bias parameter γ (third column): Group 1 only has a weak gaze bias (large γ), group 2 has a medium strong gaze bias (smaller γ), and group 3 has a very strong gaze bias (even smaller, negative γ). (TIFF) [file pone.0226428.s004.tiff]
