# Supplementary material for: GLAMbox: A Python toolbox for investigating the association between gaze allocation and decision behaviour
Source: PLoS One. 2019 Dec 16;14(12):e0226428. doi: 10.1371/journal.pone.0226428 (PMC6914332; doi:10.1371/journal.pone.0226428)
Supplement: S1 Table — The datasets are originally from Folke et al., 2017 (Experiment 2); Krajbich et al., 2010; Krajbich & Rangel, 2011 and Tavares et al., 2017 (Experiment 1). (PDF) [file pone.0226428.s005.pdf]

Dataset	N	Parameter	Mean	SD	min	25%	50%	75%	max
Folke 2016	24	v	0.25	0.08	0.17	0.19	0.22	0.3	0.51
		γ	-0.01	0.28	-0.45	-0.22	-0.04	0.2	0.43
		σ	0.19	0.03	0.12	0.17	0.19	0.21	0.28
		τ	1.85	0.97	0.43	1.19	1.62	2.52	3.92
Krajbich 2010	39	v	0.77	0.33	0.24	0.53	0.77	0.93	1.69
		γ	0.23	0.47	-0.78	-0.07	0.33	0.6	0.85
		σ	0.28	0.05	0.19	0.25	0.29	0.32	0.38
		τ	0.74	0.52	0.1	0.37	0.57	1.09	2.27
Krajbich 2011	30	v	0.67	0.33	0.18	0.44	0.63	0.78	1.58
		γ	0.19	0.41	-1.02	-0.07	0.33	0.45	0.81
		σ	0.25	0.05	0.17	0.22	0.25	0.28	0.36
		τ	1.0	0.71	0.24	0.52	0.81	1.39	3.53
Tavares 2017	25	v	0.84	0.3	0.38	0.54	0.9	1.02	1.4
		γ	0.09	0.23	-0.36	0.01	0.08	0.22	0.54
		σ	0.37	0.05	0.23	0.35	0.37	0.39	0.46
		τ	0.54	0.26	0.12	0.34	0.49	0.74	0.97

S1 Table.
