# Supplementary material for: GLAMbox: A Python toolbox for investigating the association between gaze allocation and decision behaviour
Source: PLoS One. 2019 Dec 16;14(12):e0226428. doi: 10.1371/journal.pone.0226428 (PMC6914332; doi:10.1371/journal.pone.0226428)
Supplement: Attachment — Submitted filename: Response to Reviewers.pdf [file pone.0226428.s006.pdf]

Reviewer 1

This is a very interesting study that introduces the gaze-weighted linear accumulator model (GLAM) and validates a new toolbox for fitting the model in Python to understand the association between gaze bias and decision making. In this manuscript, the authors tried to validate the GLAM and the toolbox in three different cases: individual-level parameter estimation, group-level parameter estimation using hierarchical Bayesian method, and parameter recovery. The authors show convincing and converging evidence that the GLAM performs better than a model without reflecting gaze bias in value-based decisions and explain how to use the toolbox to fit GLAM in Python. I believe the manuscript is good to be considered publication in PLOS ONE with a revision. Here, I summarize several points that I was not clear or had questions while reading it through:

1. It was not quite clear what the general speed parameter (parameter v in Eq 5, page 4) in the model captures. What does the general speed exactly mean? Does it capture speed-accuracy tradeoff as boundary parameter in DDM? It seems like Eq 6 captures accuracy-speed tradeoff using the speed parameter, but it is not quite clear for me. If the authors could provide more about this parameter, it would be better for readers to understand the parameter better.

The velocity parameter v linearly scales the item drift rates in the race process (Eq. 5) and thereby predominantly affects the response times produced by the model (lower values of v produce longer response times, whereas larger values of v produce shorter response times). It creates speed-accuracy tradeoffs in conjunction with the accumulation noise (or “diffusion”) parameter σ. This implementation is similar to other diffusion-to-bound models like the aDDM (where there is a speed parameter d and a diffusion parameter σ) or some implementations of the DDM (although some DDM parameterizations fix the diffusion parameter and estimate the boundary separation, instead).
We have added additional information about the velocity parameter v and its function in the model section:

Resulting changes in the manuscript:

ll. 110ff.
At each time step t, the amount of accumulated evidence is determined by the accumulation rate vR_i, and zero-centered normally distributed noise with standard deviation σ. The velocity parameter v linearly scales the item drift rates in the race process and thereby affects the response times produced by the model: Lower values of v produce longer response times, larger v result in shorter response times.
A choice for an item is made as soon as one accumulator reaches the decision boundary b. To avoid underdetermination of the model, either the velocity parameter v, the noise parameter σ or the decision boundary b has to be fixed. Similar to the aDDM, the GLAM fixes the decision boundary to a value of 1. [...]

2. Is Eq 4 correct? Parentheses are missing after “exp”?

We thank the reviewer for indicating this error in equation 4 of our manuscript. We have accordingly added the missing parentheses to equation 4 of our revised manuscript.

3. In Example 1, the authors collected liking scores after choice and used the liking scores to identify the higher value items or the best items. However, choice-induced preference literature has shown that choices not only reveal preferences, but also shape preferences. Thus, chosen items might have higher liking scores than non-chosen items not only because participants liked them but also because they chose them. Is there any way to rule out this issue? Or, do the authors expect different or the results if liking scores are measured in advance and test the model?

We thank the reviewer for this valuable remark. We adapted the description of this (fictitious) experiment to be more consistent with published experimental procedures, where liking ratings are collected before the choice task (e.g., Krajbich, Armel & Rangel, 2010; Krajbich & Rangel, 2011, Folke et al., 2017).

Resulting changes in the manuscript:

ll. 283ff.
While participants perform the task, their eye movements, choices and RTs are measured. Before completing the choice trials, participants were asked to indicate their liking rating for each of the items used in the choice task on a liking rating scale between 1 and 10 (with 10 indicating strong liking and 1 indicating little liking).

4. The “glam_bias.fit” and “glam_nobias.fit” lines in page 10 and page 11 do not have “chains” attribute (which is 4 in default), but the authors’ suggestion for model convergence is 2 in the main text. I found that the script in Github includes chains parameter in the script. Including the ‘chains attribute in the script examples in the manuscript would help readers more intuitively.

We thank the reviewer for pointing out that our initial manuscript was not clear enough on the number of posterior chains that we recommend for model sampling. We now explicitly include the `chains` argument in our code examples of the revised manuscript, when calling the `fit` method of the GLAM model class (see pp. 11, 12, 15, and 17).

We would further like to point the reviewer to p. 11, ll. 340 of our manuscript, where we state that the chains arguments “[...] defaults to four and should be set to at least two, in order to allow convergence diagnostics”. At least two posterior chains are needed in order to compute several common convergence diagnostic measures, such as the R-hat

measure. We recommend four chains, in accordance with the recommendations of the PyMC3 development team.

5. The authors mention about the results of model comparison test result in page 11. Without output figure or table, it was not quite easy to understand what the results look like. The Github script did not include model comparison results. Adding the model comparison result table in the revised manuscript would help readers.

We thank the reviewer for bringing this to our attention. We agree that this section was difficult to follow and have revised it substantially: The toolbox now includes a dedicated function to perform model comparisons. It wraps the PyMC3 `compare` function that was used previously, and simplifies the inputs required from the user.
We have included a paragraph describing how to perform model comparisons into the Basic Usage section of the manuscript and revised Example 1 accordingly.

Resulting changes in the manuscript:

ll. 248ff.
Comparing model variants
Model comparisons between multiple GLAM variants (e.g., full and restricted variants) can be performed using the `compare` function, which wraps the function of the same name from the PyMC3 library. The `compare` function takes as input a list of fitted model instances that are to be compared. Additional keyword arguments can be given and are passed on to PyMC3 function. This allows the user, for example, to specify the information criterion used for the comparison via the `ic` argument `'WAIC'` or `'LOO'` for Leave-One-Out cross validation). It returns a table containing an estimate of the specified information criterion, standard errors, difference to the best-fitting model, standard error of the difference, and other output variables from PyMC3 for each inputted model (and subject, if individually estimated models were given). We refer the reader to Example 2 for a usage example and exemplary output from the `compare` function.

ll. 342ff.
After convergence has been established for all parameter traces (for details on the suggested convergence criteria, see Methods), we perform a model comparison on the individual level, using the `compare_models` function from the `analysis` module (see Basic Usage: Comparing model variants):

```
comparison_df = gb.analysis.compare_models(models=[glam_bias, glam_nobias],
                                            ic='WAIC')
```

The resulting table (shown in Table 2) can be used to identify the best fitting model (indicated by the lowest WAIC score) per individual.

Table 2. Output from `compare_models` function for the first two subjects.

subject	model	WAIC	pWAIC	dWAIC	weight	SE	dSE	var_warn
0	glam_bias	523.6	5.75	0	0.94	50.25	0	0
0	glam_nobias	645.09	3.64	121.49	0.06	44.15	23.56	0
1	glam_bias	1097.86	3.69	0	1	40.32	0	0
1	glam_nobias	1185.02	2.85	87.16	0	38.22	18	0

6. Overall, I felt that the captions of figures are too long and redundant with the main text. I think it would be better to explain more in the main text and shorten the caption of figures.

We thank the reviewer for this valuable remark. In line with the reviewer's suggestion, we have shortened the (previously very long) captions of Figures 2, 6 and 7 of our revised manuscript.

Resulting changes in the manuscript:

Fig. 2. Hierarchical model structure In the hierarchical model, individual subject parameters $\gamma_i, v_i.\sigma_i$, and τ_i (subject plate) are drawn from Truncated Normal group level distributions with means μ and standard deviations σ (outside of the subject plate). Weakly informative Truncated Normal priors are placed on the group level parameters. RT and choice data $x_{i,t}$ for each trial$t$ is distributed according to the subject parameters and the GLAM likelihood (Eq (8); inner trial plate).

Fig. 6. Aggregated view of the simulated data for Example 2. (A) Mean RT binned by trial difficulty (the difference between the highest item value in a choice set and the maximum value of all others). (B) The probability that an item is chosen based on its relative value (the difference of the item's value and the maximum value of all other items in the choice set). (C) The probability of choosing an item based on its relative gaze (the difference between the gaze towards this item and the maximum gaze towards all others). (D) The probability of choosing an item based on its relative gaze, when correcting for the influence of its value. Bars correspond to the pooled data, while coloured lines indicate individual groups.

Fig 7. Pairwise comparison of posterior group-level parameter estimates between groups. Each row corresponds to one model parameter. The leftmost column shows the estimated posterior distributions for each parameter and group. Pairwise differences between the group posterior distributions are shown in all other columns. For each posterior distribution of the difference, the mean and 95% HPD are indicated, as well as the proportion of samples below and above zero (in red). All three groups

differ on the γ parameter (row B). No evidence for differences on any of the other model parameters is found (the 95% HPD of the pairwise differences between groups all include zero).

7. I could not install the toolbox using pip or conda. If the authors could make it available (or at least inform how to install on a local computer), it would help researchers access the toolbox.

We agree that the toolbox should be easily installable. We therefore packaged and distributed the toolbox on the Python Package Index (PyPi) to make it pip-installable. In addition we added a “Installation” section to the toolbox documentation (available at https://glambox.readthedocs.io), that includes instructions on installation of the toolbox and its requirements.

Minor points

8. I am not sure this is a technical issue or not, but the figures were not clear. Some letters were broken too. Please check the clarity of the figures.

We thank the reviewer for pointing this out. The figures that we have, and that we submitted to PLOS One, look clear on our computers and do not exhibit any broken letters. We formatted figures as .tiff files, as PLOS guidelines require. To resolve this issue, which seems technical to us, we have recreated all figures and again formatted to comply with PLOS guidelines. We hope that figures appear correctly now.

9. The number label in page 5 for “individual parameter estimation details” seems quite abrupt. Please drop the numbers.

We thank the reviewer for bringing this formatting error to our attention. We have removed the numbering.

Reviewer 2

Summary: this paper provides an overview of how to use the authors' toolbox to measure individual and group differences in the extent to which gaze information influences decision-making. The GLAMbox approach was first introduced in an empirical paper published this year (Thomas et al., 2019), and the current paper expands upon the method to enable other researchers to use it in an informed way. This new method is useful for researchers who use eye tracking as a tool to understand decision-making, and the GLAMbox adds a contribution to the field as a whole. Past research has focused on one overall discount value for unattended information, whereas this allows fitting of individual differences. Moreover, it seems to be a more efficient implementation than past work, which makes it more accessible. The authors are thorough in both describing model-fitting as well as parameter recovery to promote best practices. I think that a few clarifications and additions could make this paper stronger:

1. It would be helpful in the introduction and/or discussion to explain more how different individual gaze biases might arise (familiarity with items, more goal-driven approach, etc.). There is not one obvious reason, but I think some discussion of why this is important is useful. For example, Smith & Krajbich (2018) discuss "tunnel vision" as one possible mechanism.

We fully agree with the reviewer's suggestion, that adding a discussion of mechanisms underlying individual differences in the gaze bias would be interesting. We have added a paragraph in the introduction of the manuscript.

Resulting changes in the manuscript:

ll. 27ff.
Furthermore, recent findings indicate strong individual differences in the association between gaze allocation and choice behaviour (Smith & Krajbich, 2018; Thomas et al., 2019) as well as individual differences in the decision mechanisms used (Ashby et al., 2016).
While the nature of individual differences in gaze biases is still not fully understood, different mechanisms have been suggested: Smith and Krajbich (2018) showed that gaze bias differences can be related to individual differences in attentional scope ("tunnel vision"). Vaidya and Fellows (2015) found stronger gaze biases in patients with damage in dorsomedial prefrontal cortex (PFC). Further, recent empirical work has investigated the roles of learning and attitude accessibility in gaze dependent decision making (Cavanagh et al., 2019; Gwinn & Krajbich, 2020).
However, more systematic investigations of these differences are needed, as the majority of model-based investigations of the relationship between gaze allocation and choice behaviour were focused on the group level, disregarding differences between individuals.

2a: Section 0.0.1 “Individual parameter estimation details” says that the ranges chosen were derived from “sensible limits based on previous applications” (line 118). It would be helpful to have more discussion of how these sensible limits are arrived at, whether they will apply broadly to all data sets, or how to determine appropriate ranges for one’s own data including theoretical constraints.

We thank the reviewer for the suggestion to further clarify the model parameter bounds used by GLAMbox in our manuscript.

All parameter bounds were derived from an analysis of four empirical choice datasets with the GLAM (see S1 Fig and S1 Table). The estimated individual subject parameters of these four datasets, encompassing value-based and perceptual choices from up to three items (and a wide range of response times, gaze biases and choice accuracy levels), are well covered by the revised broad parameter bounds. We have revised ll. 146ff. of the manuscript to highlight this more strongly (see below).

To be certain, however, that GLAMbox can capture a wide range of possible response patterns that go beyond previous applications, we have extended the parameter bounds to cover double the range of parameters previously observed (See S1 Fig): The new ranges are:

v: [0, 4]
γ: [-2, 1]
σ: [0, 4]
τ: [0, 10]

We think that these bounds realistically cover most observable behaviour, as the v and σ parameters are naturally bound at 0, and can produce arbitrarily slow (as v approaches zero) responses and accurate (as σ approaches zero) responses.

In addition, we recommend to re-scale all item values to a range between 1 and 10, when using GLAMbox (ll. 176ff. of the revised manuscript). This, in combination with the fixed scale of gaze values ([0,1]), increases transferability of parameters between datasets. We believe that the revised parameter bounds thereby correspond to a wide range of possible response behaviours of human decision makers in simple choice tasks.

Resulting changes in the manuscript:

ll. 142ff.
The GLAM is implemented in a Bayesian framework using the Python library PyMC3 [24]. The model has four parameters (v, γ, σ, τ). By default, uninformative, uniform priors between sensible limits are placed on all parameters:

v ~ *U* (0, 4)

γ ~ *U* (-2, 1)

σ ~ *U* (0, 4)

τ ~ *U* (0, 10)

These limits were derived by extending the range of observed parameter estimates in earlier applications of the GLAM to four different empirical choice datasets. These datasets encompass data of 117 participants in value-based and perceptual choice tasks with up to three choice alternatives (including a wide range of possible response times, gaze bias strengths and choice accuracies; for further details [21]). Parameter estimates for these datasets are illustrated and summarised in S1 Table, S1 Fig and S2 Fig.

The velocity parameter *v* and the noise parameter *σ* must be strictly positive, with smaller values producing slower and more accurate responses. The gaze bias parameter *γ* has a natural upper bound at 1 (indicating no gaze bias), while decreasing *γ* values indicate an increasing gaze bias strength. The sensitivity parameter *τ* has a natural lower bound at 0 (resulting in no sensitivity to differences in average absolute decision signals $\underline{A}_i$), which larger values indicating increased sensitivity.

2b: Furthermore, the aDDM that this method seems to draw its inspiration from, uses a discount range for attention of [0-1] (Krajbich et al., 2010; Krajbich et al., 2015). However, the authors here use a range including large negative values (-10) up to 1. I think it's important to explain why negative values are used, how to interpret them (active forgetting or leaky accumulation?) and to provide a theoretical justification for their inclusion here given the context of previous literature.

We thank the reviewer for this valuable comment. We interpret negative *γ* values as indication of a leakage mechanism. For negative *γ* the sign of the absolute decision signal $\bar{A}$ (see Eq. 1) changes and evidence is actively lost for these items, when they are not fixated. This results in an overall even stronger gaze bias than for *γ* = 0 (the maximum gaze bias strength of the aDDM). Such gaze dependent leakage mechanisms are also supported by recent empirical work (see for example, Ashby et al., 2016).

To better illustrate the function of negative *γ* (and the leakage mechanism), we have extended the section on the Gaze-weighted linear accumulator model details of our revised manuscript (see below).

<u>*Resulting changes in the manuscript:*</u>

ll. 86ff.

If γ = 1, there is no difference between the biased and unbiased state, resulting in no influence of gaze allocation on choice behaviour. For γ values less than 1, the absolute decision signal A_i is discounted, resulting in generally higher choice probabilities for items that have been looked at longer. For γ values less than 0, the sign of the absolute decision signal A_i changes, when the item is not looked at, leading to an overall even stronger gaze bias, as evidence for these items is actively lost, when they are not looked at. This type of gaze-dependent leakage mechanism is supported by a variety of recent empirical findings (Ashby et al., 2016; Thomas et al., 2019).

3. The GLAM is explained in an option-wise manner. Given that recent research has shown that some individuals compare options with multiple attributes in an attribute-wise manner, would there be a way to incorporate attribute-wise comparisons into the GLAM? This may be outside the scope of the paper, but if there is a relatively easy way to implement it, that could be worth including.

We strongly agree with the reviewer that an extension to the GLAM to multi-alternative multi-attribute choice would be very interesting.
However, an extension to multiple attributes would entail changes to the format of the data that are fed into the model, and a substantial rewrite of the toolbox code.
Furthermore, how exactly attribute comparisons should be implemented specifically into the GLAM is not trivial and many possible solutions exist, which would not necessarily share code implementations: Should attribute comparisons be performed between only two alternatives? This would mean that gaze would have to be partitioned differently than only by alternatives. Comparisons between more than two alternatives could be made using an item-vs-max or item-vs-mean implementation. What happens with unattended attributes? Is their information biased or not? Would this bias be different from the alternative-wise gaze bias?
Again, we think this is a highly interesting point and we look forward to including it in a future version of GLAMbox. We think, however, that it is outside the scope of the toolbox in its current form.

Resulting changes in the manuscript:
None.

4. Figure 4 shows a strong correlation between gamma and the behavioral gaze bias. This is a good confirmation, but the behavioral measure of gaze-choice association (lines 242-246) is only very briefly mentioned. If they are so highly correlated, what does gamma add beyond the behavioral gaze bias measure? Is its main advantage including it as part of the full model estimation process?

We thank the reviewer for this valuable remark, which connects to the more general question of the contribution of a model-based analysis of behaviour vs. an analysis of purely behavioural measures (such as our behavioural gaze bias measure). We believe

that there are several key differences between our behavioural gaze bias measure and our model-based analysis of individuals' gaze bias strength (as quantified by the γ parameter): First, the model-based analysis poses several strict assumptions on the data generation process, by explicitly stating how we assume gaze to be involved in the decision process. The behavioural measure, on the other hand, does not include such constraints, as it purely quantifies the correlation between gaze allocation and choice, when corrected for the items' values. If we find that the model describes the data well, we therefore have more evidence for the data generating mechanism, when compared to our purely behavioural measure. Second, by fitting a model to the data, we are able to make out-of-sample choice and response time predictions and compare these to the empirical data, thereby allowing for a more rigorous test of the fit of the model to the data. Lastly, a model-based analysis also allows for a comparison of different decision processes through a likelihood-based model comparison analysis.

5. Example 2: does it make sense to expect similar parameter ranges for patients compared to a young, healthy sample? Parameters such as noise might be higher and drift rate might be slower. I don't think that this should affect the gaze bias estimation, but it might affect which parameters are used to constrain hierarchical estimation.

We thank the reviewer for the comment. We are not sure, however, if we understand it correctly. Indeed, in Example 2, we simulate an experiment with three fictitious patient groups, that only differ on their gaze bias parameter, and not on other model parameters, such as the velocity parameter v or the accumulation noise σ. We think that this scenario is not unrealistic. For example, the study that we modeled this example after (Vaidya & Fellows, 2015) did not find systematic differences between different patient groups and healthy controls on integration speed or the threshold parameter controlling speed accuracy tradeoffs.
We agree with the reviewer, however, that in other settings different groups (e.g., patients and healthy controls) can of course differ on more than just the gaze bias parameter. This is why the model that is used in this example contains group-dependencies on all model parameters, not just the gaze bias parameter γ (specified using the `depends_on` keyword). In the resulting model, individual estimates from one subject are informed by all other subjects *in the same group*, and group-level estimates are obtained per group. However, no hierarchical structure across groups is built for a parameter, when it is specified as having a group-dependency. If a researcher wishes a parameter to be estimated across groups, then she can opt to not list dependencies for this parameter. We revised the manuscript to better communicate these details to the reader.

Resulting changes in the manuscript:

ll. 411ff.

We simulate data of three patient groups (N_1 = 5, N_2 = 10, N_3 = 15), with 50 trials per individual, in a simple three item value-based choice task, where participants are instructed to simply choose the item they like the best. These numbers are roughly based on a recent clinical study on the role of the prefrontal cortex in fixation-dependent value representations (Vaidya & Fellows, 2015). Here, the authors found no systematic differences between frontal lobe patients and controls on integration speed or the decision threshold, controlling speed-accuracy trade-offs. Therefore, in our example we only let the gaze bias parameter γ differ systematically between the groups, with means of $\gamma = 0.7$ (weak gaze bias), $\gamma = 0.1$ (moderate gaze bias) and $\gamma = -0.5$ (strong gaze bias), respectively. We do not assume any other systematic differences between the groups and sample all other model parameters from the estimates obtained from fitting the model to the data of Krajbich and Rangel (2011) (for an overview of the generating parameters, see S4 Fig).

ll. 443ff.
In this model, each parameter is set up hierarchically within each group, so that individual estimates are informed by other individuals in the same group. If the researcher does not expect group differences on a parameter, this parameter can simply be omitted from the `depends_on` dictionary. The resulting model would then assume that all parameter estimates of all individuals (across all groups) come from the same group-level distribution.

6. In Fig. 6, choice difficulty is defined as the highest value is compared with the average of the other values. However, I think a choice would be more difficult if the second highest value were quite similar to the highest value, regardless of the lower value options. For example, a choice with two similarly high value options and one very low value option would be harder than one with one high value option and 2 medium value options, but both would be similar difficulty by the metric currently used. Is there a reason this is favored over comparing the best and next best options?

We thank the reviewer for this insightful remark. We agree that the difference between the highest item value and the second highest value is a more common and intuitive measure of choice difficulty than the difference to the mean. For this reason, we have adapted the choice difficulty measure of Figure 6A accordingly. We have also adapted the relative item value and gaze measures in Figure 6B-D to follow the same comparison strategy, by always computing the difference between an item's value / gaze and the maximum of all others.

Resulting changes in the manuscript:
Figure 6 (and caption)

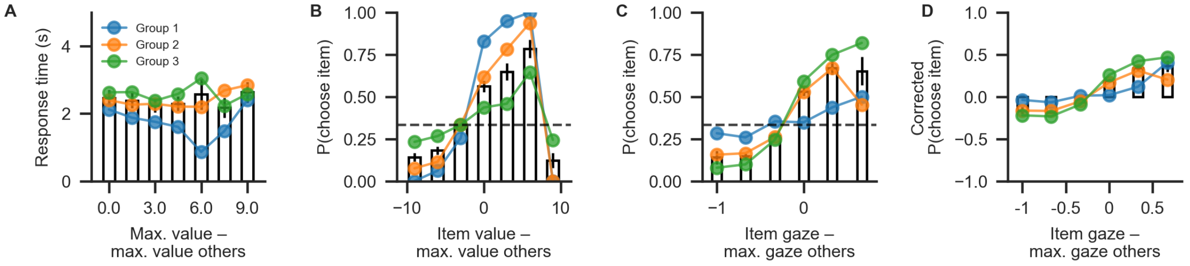

7. A different number of draws and burn in samples are used in model-fitting from Example 1 to Example 2; is there a reason for this? Perhaps briefly explain why if it is relevant to users.

We thank the reviewer for raising this point. We agree that the different numbers should be justified. The reason to increase the number of burn-in and kept samples is a higher autocorrelation of samples from the hierarchical model, which contains many more parameters than individual models. To obtain enough effective samples (Kruschke, 2014) for each parameter, the total number of samples is increased. We have added a paragraph and corresponding references to the manuscript.
We also changed the number of burn-in samples and samples to keep to 20.000 for this model.

Resulting changes in the manuscript:

ll. 450ff.
After the model is built, the next step is to perform statistical inference over its parameters. As we have done with the individual models, we can use MCMC to approximate the parameters' posterior distributions (see Methods for details). Due to the more complex model structure and drastically increased number of parameters, the chains from the hierarchical model usually have higher levels of autocorrelation. To still obtain a reasonable number of effective samples (Kruschke, 2014), we increase the number of tuning- and draw steps:

```
hglam.fit(method='MCMC',
          draws=20000,
          tune=20000,
          chains=4)
```

Small clarifications/phrasing corrections:

8 • In the abstract, I would rephrase the middle sentence beginning with “However, only few decision models exist…” to something like, “However, few decision models exist that enable a straightforward characterization of the gaze-choice association at the individual level…”

We thank the reviewer for this helpful suggestion. We have changed the referenced sentence in the abstract of our manuscript accordingly.

Resulting changes in the manuscript:

(Abstract)
However, few decision models exist that enable a straightforward characterization of the gaze-choice association at the individual level, due to the high cost of developing and implementing them.

9. In the introduction line 4, “It was repeatedly shown” should be changed to “It has been repeatedly shown”

Thank you for pointing this out.

Resulting changes in the manuscript:

ll. 3f.
For example, in value-based decision making, it has been repeatedly shown that longer gaze towards one option is associated [...]

10. Line 66, “i” is not explained. It can be inferred that it indexes each item, but it should be explicitly mentioned.

We thank the reviewer for pointing out the missing clarification of the *i* index used in the Gaze-weighted linear accumulator model details section. It indeed indexes the items in a choice set. We have added a clarification as follows:

Resulting changes in the manuscript:

ll. 80ff.
Throughout the trial, the absolute signal of an item *i* can be in two states: An unbiased state, equal to the item’s value r_i while the item is looked at, and a biased state while any other item is looked at, where the item value r_i is discounted by a parameter γ.

11. Figure 1 and equation 2 (lines 76-77). What does the “maximum of all other decision signals mean”? The highest average absolute decision signal among the item options? My

interpretation is that you are subtracting the highest value option from all others as a sort of normalization, but this isn't quite coming through clearly.

We thank the reviewer for the comment. We agree that this section was unclear. We have reworded it to be more specific about the arithmetic operations performed. We have also revised the notation in the equation, changing *J* to "$j \neq i$" to be more explicit about the group of variables that the maximum operator entails (also see next point).

Resulting changes in the manuscript:

ll. 93ff.
To determine the relative decision signals, the average absolute decision signals $\underline{A}_i$ are transformed in two steps: First, for each item *i*, the relative evidence R_i^* is computed as the difference between the average absolute decision signal of the item $\underline{A}_i$ (Eq. 1) and the maximum of all other average absolute decision signals $\underline{A}_{j \neq i}$ (also obtained from Eq. 2) is computed [...]

12. Line 76, equation 2. What does J represent? From reading the empirical paper using the same method, Thomas et al., 2019, it sounds like J represents the set of all items, but it should also be defined in this paper.

We thank the reviewer for pointing out the missing clarification of the *J* index used in the Gaze-weighted linear accumulator model details section. In its current form, *J* represents the set of all the items in a choice set except for item *i*.

To avoid any further confusion, we have adapted Eq 2 and 8, by directly specifying $j \neq i$. (also see previous point).

Resulting changes in the manuscript:

ll. 93ff.
To determine the relative decision signals, the average absolute decision signals $\underline{A}_i$ are transformed in two steps: First, for each item *i*, the relative evidence R_i^* is computed as the difference between the average absolute decision signal of the item $\underline{A}_i$ (Eq. 1) and the maximum of all other average absolute decision signals $\underline{A}_{j \neq i}$ (also obtained from Eq. 2) is computed [...]

ll. 125ff.
Hence, the joint probability $p(t)_i$ that accumulator E_i crosses *b* at time *t*, and that no other accumulator $E_{j \neq i}$ has reached *b* first, is given by:

13. Figure 1e is above panel d in a way that violates expectations of reading/processing material, and I think it would be clearer if the panel positions for d and e were switched (even though I understand it was likely put there for design reasons).

We have changed the panel labeling and figure caption so that labels only go from left to right.

Resulting changes in the manuscript:
Figure 1 (including caption)

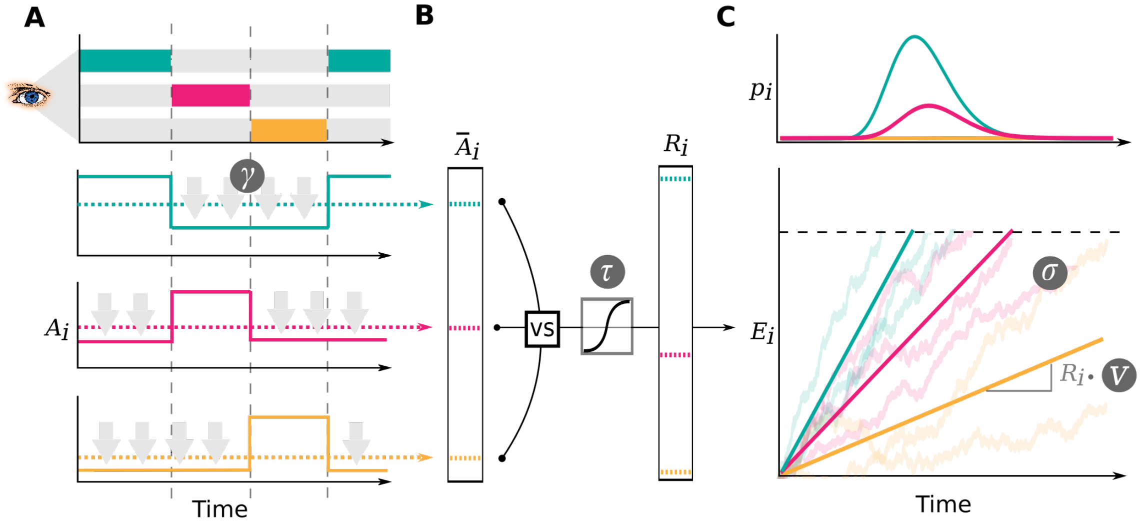

14. Figure 3 flips the orientation of the axes between D, E, and F so that the same variables are on the x versus y axes, which makes it harder to process them all at once. It would better fit your description for “gaze influence on choice” to be on the x-axis in F. I realize that these are non-directional correlations and that the axes may be flipped to better align with the above histograms, but I find it harder to parse this way (instead of just including the histogram distributions with their own separate x-axis labels).

We thank the reviewer for this valuable suggestion. Unfortunately, it is, to our knowledge, not possible to plot the same variable on the x-axis of all three pairwise correlation panels of Figure 3. To make the figure more consistent, however, we adapted Figure 3 to plot the variables in the following configuration (notation: panel: x-axis, y-axis):

- **D**: P(choose best), Mean RT
- **E**: Gaze influence on P(choice | value), Mean RT
- **F**: Gaze influence on P(choice | value), P(choose best).

In this new configuration, panels D and E share the same y-axis (Mean RT), while panels E and F share the same x-axis (Gaze influence on P(choice | value)).

As a result of this change, we have also detached the marginal histograms (panels A-C) from the pairwise correlation plots (panels D-E). The histograms are now plotted independently.

Resulting changes in the manuscript:
Figure 3 (including caption)

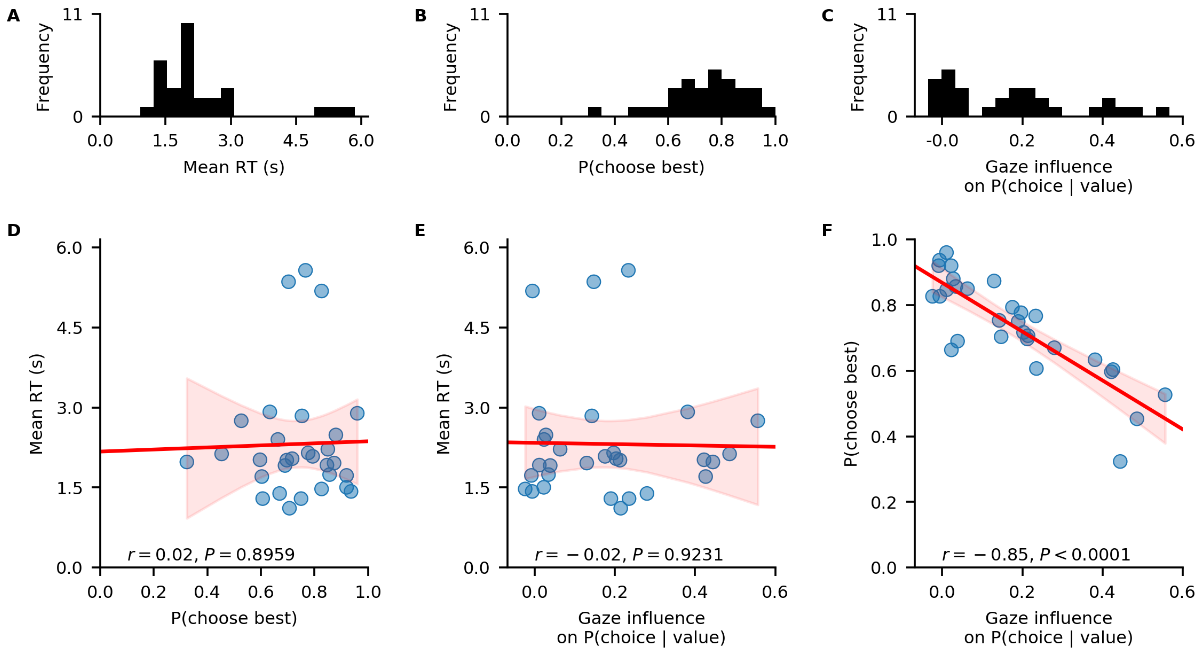

15. Figure 5, I might put “simulated observed” instead of just “observed” on the x-axis to make sure that readers don’t get confused and think that the data is actual raw data rather than data simulated from inputted parameters. Alternatively you could mention it in the figure caption.

We thank the reviewer for this suggestion. We agree that this is more transparent and reduces risk of confusion. We have adapted the axis label and figure caption.

Resulting changes in the manuscript:

Figure 5 caption and axis labels:
Comparison of individuals' simulated observed response behaviour with the out-of-sample predictions of a GLAM variant [...]

Reviewer 3

This study presents a python toolbox to fit parameters for the authors' gaze-weighted linear accumulator model, capitalizing on python's Bayesian package PyMC3. Fitting a DDM is quite computationally complex, with many researchers who are interested in the theory perhaps not having the skills required to write their own estimation code. Moreover, it is typically a very time-consuming process to fit these kinds of models, so a faster methods are always welcome additions. They use a race model which can handle non-binary choices will help better approximate real-world settings.
I really like that they have included parameter recovery into their toolbox. In addition, doing model comparison with and without the gaze bias parameter is nice – particularly as it can help other researchers understand under which situations gaze is and is not important.

Some guidance on what to do to compare multiple conditions/tasks would be a nice feature

We thank the reviewer for pointing this out. We agree that this would be an important feature of a toolbox. We have therefore included an additional subsection to the "Basic usage" section of the manuscript, detailing the toolbox's functionality to perform and visualize Bayesian parameter comparisons between multiple groups (like in Example 2) or conditions (which was previously not documented).
We have also explicitly included a similar section that describes how to perform comparisons between different model variants.

Resulting changes to the manuscript:

ll. 229ff.
Comparing parameters between groups or conditions
Parameter estimates can be compared between different experimental groups or conditions (specified with the `depends_on` keyword when calling `make_model`) using the `compare_parameters` function from the `analysis` module. It takes as input the fitted GLAM instance, a list of parameters (`'v'`, `'s'`, `'gamma'`, `'tau'`), and a list of pairwise comparisons between groups or conditions. The comparison argument expects a list of tuples (e.g., `[('group1', 'group2'), ('group1', 'group3')]`). For example, given a fitted model instance (here `glam`) a comparison of the γ parameter between two groups (`group1` and `group2`) can be computed as:

```
from gb.analysis import compare_parameters
comparison = compare_parameters(model=glam,
                                parameters=['gamma'],
                                comparisons=[('group1', 'group2')])
```

The function then returns a table with one row per specified comparison, and columns containing the mean posterior difference, percentage of the posterior above zero, and corresponding 95% HPD interval. If supplied with a hierarchical model, the function computes differences between group-level parameters. If an individual type model is given, it returns comparison statistics for each individual.
Comparisons can be visualized using the `compare_parameters` function from the `plots` module. It takes the same input as its analogue in the `analysis` module. It plots posterior distributions of parameters and the posterior distributions of any differences specified using the `comparisons` argument. For a usage example and plot see Example 2 and Fig. 7.

Comparing model variants
Model comparisons between multiple GLAM variants (e.g., full and restricted variants) can be performed using the `compare_models` function, which wraps the function of the same name from the PyMC3 library. The `compare_models` function takes as input a list of fitted model instances that are to be compared. Additional keyword arguments can be given and are passed on to the underlying PyMC3 `compare` function. This allows the user, for example, to specify the information criterion used for the comparison via the `ic` argument (`'WAIC'` or `'LOO'` for Leave-One-Out cross validation). It returns a table containing an estimate of the specified information criterion, standard errors, difference to the best-fitting model, standard error of the difference, and other output variables from PyMC3 for each inputted model (and subject, if individually estimated models were given). We refer the reader to Example 1 for a usage example and exemplary output from the `compare_models` function.

In addition, I think the github documentation needs more details and guidance (e.g., simply to tell the reader to use Jupyter to open the readme). In addition, I ran into some errors using the code, which could have been the result of poor documentation.

We thank the reviewer for pointing out that our previous documentation of GLAMbox was not sufficient. To make GLAMbox more accessible for the reader, we have created a self-contained documentation page of the toolbox (including “Installation”, “Quickstart”, “Basic Usage” and the use case examples, which can now be viewed comfortably in a browser, without the need for a running python installation or jupyter). This documentation is now explicitly referenced in the abstract and can be found at https://glambox.readthedocs.io. The documentation now states explicitly that the notebooks can be run interactively by opening them with the Jupyter software. We also extended the example notebooks by including text from the manuscript in them to better guide the reader. The thereby notebooks now act as standalone tutorials for GLAMbox. The documentation also contains full API reference of all functions and methods available to the user.

I have the following suggestions/issues:

I would like to point the authors to Smith, Krajbich, and Webb (Estimating the dynamic role of attention via random utility – 2019) which estimates aDDM's theta parameter using a very fast and simple regression method, which seems relevant to their work.

We thank the reviewer for this comment. We have added a paragraph to the Discussion where we discuss other existing approaches to obtaining gaze bias estimates, highlight differences and commonalities between the GLAM and other approaches.

Resulting changes in the manuscript:

ll. 544ff.
The goal of GLAM is to provide a model-based estimate of the gaze bias on the level of an individual (as indicated by GLAM's γ parameter), in choice situations involving more than two choice alternatives. To estimate the gaze bias, GLAM describes the decision process in the form of a linear stochastic race and aggregates over the specific sequence of fixations during the decision process (by only utilizing the fraction of the decision time that each item was looked at). These two characteristics distinguish the GLAM from other existing approaches of obtaining an estimate of individuals' gaze bias:

First, the GLAM is focused on quantifying the gaze bias on the individual level. It does not capture dynamics of the decision process on the level of single fixations. If these fine-grained dynamics are of interest to the researcher, the aDDM can be used. Here, the fixation-dependent changes in evidence accumulation rates throughout the trial are not averaged out. Keeping this level of detail, however, comes at a cost: Fitting the aDDM relies on extensive model simulations (including a simulation of the fixation process; for a more detailed discussion see Thomas et al., 2019). The GLAM, on the other hand, aggregates over the fixation-dependent changes in the accumulator's drift rate in order to simplify the estimation process of the gaze bias.

Second, the GLAM directly applies to choice situations involving more than two choice alternatives. While the GLAM has been shown to also capture individuals' gaze bias and choice behaviour well in two-alternative choice situations \cite{thomas_gaze_2019}, there exist other computational approaches that can estimate the gaze bias of an individual in binary decisions: If response times are of interest to the researcher, the gaze bias can be estimated in the form of a gaze-weighted DDM (see for example Cavanagh et al., 2014, Lopez-Persem et al., 2016). Similar to the GLAM, this approach also aggregates over the dynamics of the fixation process within a trial, by only utilizing the fraction of trial time that each item was looked at. In contrast to the GLAM, however, gaze-weighted DDM approaches describe the decision process in the form of a single accumulator that evolves between two decision bounds (each representing one of the two choice alternatives). For two-alternative choice scenarios, where response times are not of interest to the researcher, Smith and colleagues (2019) proposed a method of estimating the aDDM gaze-bias parameter through a random utility model. Here, the gaze bias can be estimated in a simple logit model.

In addition, it would be nice to see a discussion/comparison of this to other race models (an unacquainted reader may incorrectly believe that theirs is the first race model to vit ddm-eqsue parameters upon reading their introduction), as well as a discussion of the drawbacks of race models relative to more traditional aDDM methods.
Although this reviewer is familiar with the authors' previous work on the GLAM model, it may be useful to have a section with more comprehensive introduction to the model/theory and comparison to similar models like the aDDM (subject to editorial guidance – I am not sure what is appropriate).

We thank the reviewer for the comment. We have revised portions of the introduction and discussion sections to characterise and contextualize the GLAM more appropriately. We now state explicitly that the GLAM builds on other existing race models and discuss drawbacks and benefits of choosing the race framework. Specifically, we state that, while it has been shown that race models do not necessarily perform optimally (Bogacz et al., 2006), they can be more efficient in application (as analytical solutions to their first-passage density exist), they naturally extend to scenarios with more than two items, and we could show previously that they capture empirical data well (Thomas et al., 2019).
The manuscript was also revised to better position the GLAM more clearly in contrast to other existing models for gaze-dependent choices (e.g., the aDDM, gaze-weighted DDM approaches) (see previous response).

Resulting changes to the manuscript:

ll. 39ff.
With the Gaze-weighted linear accumulator model (GLAM; Thomas et al., 2019), we have proposed an analytical tool that allows the model-based investigation of the relationship between gaze allocation and choice behaviour at the level of the individual, in choice situations involving more than two alternatives, solely requiring participants' choice, response time (RT) and gaze data, in addition to estimates of the items' values.

Like the attentional Drift Diffusion Model (aDDM; Krajbich et al., 2010; Krajbich & Rangel, 2011; Krajbich et al, 2012), the GLAM assumes that the decision process is biased by momentary gaze behaviour: While an item is not fixated, its value representation is discounted. The GLAM, however, differs from the aDDM in other important aspects: In contrast to the aDDM, the fixation-dependent value signals are averaged across the trial, using the relative amount of time individuals spend fixating the items. This step abstracts away the specific sequence of fixations in a trial, that can be investigated with the aDDM. This simplification allows for the construction of constant drift rates in a trial that can enter a basic linear stochastic race framework. While race models like the GLAM are not statistically optimal (Bogacz et al., 2006) the GLAM has been shown to provide a good fit to empirical data (Thomas et al., 2019). In general,

race models have at least two practical advantages: First, they often have analytical solutions to their first-passage density distributions, and secondly, they naturally generalize to choice scenarios involving more than two alternatives. The analytical tractability of the race framework further allows for efficient parameter estimation in a hierarchical Bayesian manner.
The GLAM thereby combines gaze-dependent accumulation with the computational advantages of linear stochastic race models.

ll. 77f.
Like the aDDM, the GLAM assumes that preference formation, during a simple choice process, is guided by the allocation of visual gaze (for an overview, see Fig. 1).

ll. 106ff.
Unlike more traditional diffusion models (including the aDDM), the GLAM employs a linear stochastic race to capture response behaviour as well as RTs. The relative signals R_i enter a linear stochastic race, where one item accumulator E_i is defined for each item in the choice set:

A much more extensive readme file and instructions should be included. For example, this reviewer know that the examples/readme are to be opened in jupyter, but some (nay, many – esp. those searching for a toolbox rather than writing their own code) may not. A basic guide for others would be helpful.

We have created a self-contained documentation page for the toolbox, which can be found at https://glambox.readthedocs.io. It now includes more information, including installation instructions, a “Quickstart” section, full API reference of all the available functions, and a more extensive section on “Basic Usage”. The documentation also includes rendered versions of the Example notebook files (that can be directly viewed in the browser) and information on how to run the notebooks interactively.

When I attempted to run the parameter recovery exercise, I received an error originating in glam.fit (AttributeError: Can't pickle local object 'make_subject_model..lda_logp'), but don’t know whether that was my poor execution or a problem in the code.

We cannot reproduce the issue on multiple machines under different operating systems. We suspect the issue to be an older version of PyMC3 (<3.7). To further investigate the issue, we would be glad if the reviewer could share information on their Python environment (Python version, version of installed packages).
We hope that the PyPi packaged version of the toolbox and our more detailed installation guide will help prevent such errors in the future.
